# Real-life clinical pattern, management, and survival in Thai patients with early-stage or metastatic triple-negative breast cancer

**DOI:** 10.1371/journal.pone.0209040

**Published:** 2018-12-19

**Authors:** Vichien Srimuninnimit, Piti Pornpraserthsuk, Arkom Chaiwerawattana, Youwanush Kongdan, Teerayuth Namkanisorn, Areewan Somwangprasert, Chulaporn Jatuparisuthi, Puttisak Puttawibul, Mawin Vongsaisuwan, Luangyot Thongthieang, Chanyoot Bandidwattanawong, Chaturong Tantimongkolsuk

**Affiliations:** 1 Department of Medicine, Siriraj Hospital, Bangkok, Thailand; 2 Chemotherapy Unit, Lopburi Cancer Hospital, Lopburi, Thailand; 3 National Cancer Institute, Bangkok, Thailand; 4 Department of Surgery, Ramathibodi Hospital, Bangkok, Thailand; 5 Department of Medicine, Prapokklao Hospital, Chanthaburi, Thailand; 6 Department of Surgery, Maharaj Nakorn Chiang Mai Hospital, Chiang Mai, Thailand; 7 Department of Medicine, Klang Hospital, Bangkok, Thailand; 8 Department of Surgery, Songklanagarind Hospital, Songkla, Thailand; 9 Department of Surgery, King Chulalongkorn Memorial Hospital, Bangkok, Thailand; 10 Department of Medicine, Khon Kaen Hospital, Khon Kaen, Thailand; 11 Department of Medicine, Vajira Hospital, Navamindradhiraj University, Bangkok, Thailand; 12 Department of Medicine, Somdech Phra Pinklao Hospital, Bangkok, Thailand; Florida State University, UNITED STATES

## Abstract

**Objectives:**

To characterize the clinical pattern and evaluate real-life practices in the management of patients with triple-negative breast cancer (TNBC) in Thailand.

**Methods:**

In this multicenter, prospective, observational cohort, females (aged ≥18 years) with histologically and immunohistochemically confirmed TNBC were enrolled. Patient data was collected at four study visits–an inclusion visit (for enrollment), and three subsequent follow-up visits at 12±1, 24±1, and 36±1 months after completion of first day of any planned chemotherapy.

**Results:**

Of the 293 enrolled patients, 262 (89.4%) had early-stage TNBC (Stage I: 46 patients, Stage II: 151 patients, and Stage III: 65 patients) and 31 (10.6%) had metastatic TNBC (mTNBC). Chemotherapy was prescribed to 95.4% of the early-stage patients and to 100.0% of the mTNBC patients; most commonly as anthracycline-based in combination with cyclophosphamide and other agents. Patients’ performance status and consensus guidelines were the major factors affecting choice of treatment. In early-stage patients, median disease-free survival (DFS) and overall survival (OS) had not been reached for Stage I and II patients, and were calculated to be 37.0 months and 40.0 months, respectively, in Stage III patients. In mTNBC patients, progression-free survival (PFS) and OS were found to be 10.0 months and 14.0 months, respectively. In Stage III patients, anthracycline-based regimens were found to be associated with increase in DFS (p = 0.0181) and OS (p = 0.0027) compared to non-anthracycline-based regimens. In mTNBC patients, non-taxane-based regimens were associated with an increase in PFS (p = 0.0025). The 3-year survival rates in early-stage and mTNBC patients were 85.0% and 21.0%, respectively.

**Conclusion:**

Clinical management of TNBC in Thailand follows the general guidelines for treatment of TNBC. However, prognosis and survival outcomes are suboptimal, especially in progressive disease. This study is the first assessment in the existing practices in which the results could pave to way to improve the treatment outcome of TNBC in Thailand.

## Introduction

Triple-negative breast cancer (TNBC) is a breast cancer phenotype characterized by an immunohistochemical absence of estrogen receptor (ER) and progesterone receptor (PR), as well as absence of amplification of the human epidermal growth factor receptor 2 (HER2) gene [1]. Due to tumor heterogeneity and presence of several subtypes, TNBC is of particular concern; it is clinically aggressive compared to other breast cancers, has poor prognosis on overall survival (OS) and disease-free survival (DFS), lacks specific target for therapeutic intervention, and overlaps with BRCA1-associated breast cancers. [2] Approximately 10–20% of all breast cancers are TNBC [3–5] and the prevalence is high in Asians [6, 7]. Data on the prevalence of TNBC in Thailand is sparse but in a recent study on survival time in breast cancers of different molecular subtypes, >30.0% of study patients were found to have TNBC [8]. There has been an increasing incidence of breast cancer in Thailand in recent years [9, 10] and this indicates that TNBC-related morbidity and mortality will certainly rise in the future.

Currently the mainstay of TNBC treatment is surgery with or without adjuvant chemotherapy [11] and the choice of therapeutic approach depends on the level of advancement of the disease. Early-stage TNBC is shown to have high sensitivity to adjuvant chemotherapy [12] and is associated with an improvement in DFS and OS [13]. In these patients, depending on their performance status, anthracycline- and taxane-based regimens are the standard of care while docetaxel in combination with cyclophosphamide is considered as an acceptable non-anthracycline-based adjuvant chemotherapy [13–15]. On the other hand, treatment of metastatic TNBC (mTNBC) follows the general principles for all metastatic breast cancers and is largely palliative instead of curative [16]. The main goal of chemotherapy in mTNBC is to prolong progression-free survival (PFS) and OS as well as to improve patients’ quality of life. Currently, there is no first-line standard of care for treatment for mTNBC, and due to the lack of clinical success of targeted therapies, the most common approach involves the use of taxanes, anthracyclines, vinca alkaloids, and antimetabolites, either as a single-agent or multiple agents in combination or sequential manner [1, 17].

In order to implement evidence-based strategies in cancer medicine and introduce newer therapies and regimens, it is necessary to first assess the existing standard practices and identify any shortcomings in these. Hence, the primary objective of the study was to characterize clinical patterns of newly diagnosed TNBC patients in Thailand in real-life practice. Secondary objectives included identification of existing treatment patterns and assessment of 3-year survival rates in study patients.

## Materials and methods

### Study design

The reported study was a national, multicenter, non-interventional, prospective cohort analysis with a three-year follow-up.

The study included four patient visits over three years–an inclusion visit (V0) during which the patient was enrolled, and subsequent yearly visits (V1–V3) at 12 ± 1 months, 24 ± 1 months, and 36 ± 1 months after completion of first day of chemotherapy.

The study was performed in accordance with the principles laid down by the 18^th^ World Medical Assembly (Helsinki 1964) and all subsequent amendments. Moreover, the study conduct was in compliance with the guidelines for good epidemiological practice, STROBE guidelines for reporting observational studies as well as national and local laws and regulations in Thailand. The study protocol has been approved by local Ethics Committees of 16 study sites (where some study site has shared the same Ethics Committees) including Siriraj Institutional Review Board; Human Research and Ethics Committee, Lopburi Cancer Hospital; Research Committee, National Cancer Institute; Committee on Human Rights Related to Research Involving Human Subjects, Ramathibodi Hospital; Ethics Committee, Prapokklao Hospital; Research Ethics Committee, Chiang Mai University; Ethics Committee For Researche Involving Human Subjects, the Bangkok Metropolitan Administration; Research Ethics Committee, Prince of Songkla University; Institution Review Board, Chulalongkorn University; Human Research Ethics Committee, Khon Kaen Hospital; Research Ethics Committee, Vajira Hospital; Human Research and Ethics Committee, Naval Medical Department; Institutional Review Board, Royal Thai Army Medical Department. All study patients had to provide a signed and dated informed consent.

### Patient recruitment and data collection

Patient recruitment for the study began in July 2011 and the last patient finished their last follow-up visit in January 2016.

Study patients were females (aged ≥18 years) with histologically documented TNBC fulfilling the following criteria: 1) ER negative (ER–ve), and PR negative (PR–ve), defined by <1% tumor staining by immunohistochemistry (IHC), and 2) HER2 non-overexpression, defined by IHC score of (0,1) or fluorescence *in situ* hybridization-negative (FISH–ve), or silver *in situ* hybridization-negative (SISH–ve). In addition, study patients were not to have received prior chemotherapy for their TNBC (adjuvant or neoadjuvant chemotherapy for early-stage TNBC or chemotherapy for metastatic TNBC). Patients presenting with *in situ* breast carcinoma, or were non-TNBC, or had a concurrent history of other neoplasm (except curatively treated basal cell skin cancer or adequately treated *in situ* cervical carcinoma) were excluded from the study.

Data collected at baseline (V0) included patient demographics, relevant medical history, circumstances of detection, tumor characteristics, TNM staging (AJCC 7^th^ Ed), treatments–prior and planned, factors influencing planned treatment (classified into tumor factor, subject factor, and center factor). Data collected at V1–V3 included performance status according to Eastern Cooperative Oncology Group (ECOG) scores, treatments received, treatments ongoing and reasons for change in planned treatment (only V1), date and pattern of recurrence, and patient survival status–OS, DFS, and PFS.

### Statistical analysis

Calculation of sample size was made after taking in consideration the national incidence in 2010 in Thailand of 13,310 new breast cancer cases, of which 11% (1471 cases) were estimated to be TNBC. In order to enroll approximately 20% of the annual TNBC cases and allowing for a 10% annual dropout rate, a total of 300 TNBC patients were targeted as the sample size for enrolment into the study.

### Statistical methods

Study variables were analyzed using descriptive statistics–mean, standard deviation (SD), median, range, and 1^st^ and 3^rd^ quartiles for continuous parameters, and counts and percentages for categorical parameters. Survival curves were designed using Kaplan-Meier method and stratified by ECOG scores and tumor burden. Statistical significance between chemotherapy regimens was calculated using log-rank test. All analyses were performed with SAS version 9.4 (SAS Institute, Cary, North Carolina).

### Subgroup and post hoc analyses

Certain subgroup analyses were carried out to ascertain the effects of anthracycline and taxane regimens; specifically, taxane vs. non-taxane, and anthracycline vs. non-anthracycline. Moreover, *post hoc* analyses were carried out to assess the effect of taxane add-on to anthracycline-based adjuvant chemotherapy in Stage III patients. Data were analyzed for median follow-up period, median DFS and OS, as well as for survival probability at one, two, and three years following enrollment.

## Results

### Patient disposition and characteristics

A total of 293 patients enrolled at 16 sites of whom 262 had early-stage TNBC and 31 had mTNBC. In the early-stage patients the Tumor-Node-Metastasis staging classified 46, 151, and 65 patients in Stage I, Stage II, and Stage III, respectively. A total of 198 (67.6%) patients completed the study while 61 (20.8%) patients died and 24 (8.2%) patients were lost to follow-up. Ten (3.4%) patients withdrew consent during the course of the study. Of the 198 patients who completed the study, 187 (94.4%) were alive without recurrent disease at the final visit.

Median ages of early-stage and mTNBC patients were 53.0 [inter-quartile range (IQR): 44.0–60.0] and 56.0 (IQR: 47.0–61.0) years, respectively Table 1, and 60.4% of patients were aged 41–60 years. At enrollment, 92.1% (n = 270) of patients had ECOG = 0. A total of 25 (8.5%) patients had a prior history of breast cancer, and this proportion was much higher in patients with mTNBC in comparison to patients with early-stage TNBC (48.4% [n/N = 15/31] vs. 3.8% [n/N = 10/262]). The proportion of patients with a family history of breast cancer and ovarian cancer was 8.2% (n = 24) and 1.4% (n = 4), respectively. Over half (n = 177, 60.4%) of the study patients were postmenopausal.

**Table 1:** Performance status and medical history of enrolled patients.

	Early-stage	Metastatic
	N = 262	N = 31
Age in years,		
Mean (SD)	52.42 (12.0)	54.87 (9.8)
Median (IQR)	53.0 (44.0–60.0)	56.0 (47.0–61.0)
ECOG Performance Status		
0	247 (94.3)	23 (74.2)
1–2	15 (5.7)	8 (25.8)
Family history		
Breast cancer	19 (7.3)	5 (16.1)
Ovarian cancer	3 (1.2)	1 (3.2)
Menopausal status		
Premenopausal	90 (34.4)	4 (12.9)
Perimenopausal	20 (7.6)	2 (6.5)
Postmenopausal	152 (58.0)	25 (80.7)
Histopathological grade at diagnosis^a^		
Not assessable	9 (3.4)	14 (45.2)
Well differentiated	13 (5.0)	0 (0.0)
Moderately differentiated	108 (41.2)	6 (19.4)
Poorly differentiated	130 (49.6)	11 (35.5)
Breast cancer surgery		
Prior surgery	240 (91.6)	22 (71.0)
Partial mastectomy	29 (11.1)	1 (3.2)
Total mastectomy	206 (78.6)	20 (64.5)
Nodal status		
Nodal excision	216 (82.4)	21 (67.7)
Number of nodes excised, median (IQR)	16.0 (11.0–21.0)	12.0 (10.0–19.0)
Number of nodes positive, median (IQR)	0.0 (0.0–0.0)	2.0 (0.0–10.0)

IQR–interquartile range; ECOG–Eastern Cooperative Oncology Group. Values are presented as n (%) unless specified otherwise

^a^ Data for 2 patients in the early-stage group was missing in diagnosis

The commonly employed methods for breast cancer detection were core biopsy, excisional biopsy, and diagnostic mammogram, employed in 41.8% (n = 122), 31.5% (n = 92), and 18.8% (n = 55), of the patients, respectively.

All patients (N = 293) were diagnosed with ductal carcinoma. Overall, TNBC tumors were moderately to poorly differentiated Table 1. In early-stage patients, tumors were mostly poorly differentiated (49.6%, n/N = 130/262) or moderately differentiated (41.2%, n/N = 108/262). In patients with mTNBC, the majority of the tumors were either not assessable (45.2%, n/N = 14/31) or poorly differentiated (35.5%, n/N = 11/31).The most common sites of metastasis in patients with mTNBC were lungs (n = 13), liver (n = 6), and bones (n = 6).

Almost 90.0% (n/N = 262/293) of the patients had undergone breast surgery prior to enrollment, the majority (77.1%, n = 226) of which was total mastectomy Table 1. Mean number of nodes excised and nodes positive for malignancy were 16.5 (±8.1) and 2.8 (±5.6), respectively. Eight (25.2%) patients with mTNBC received radiation therapy prior to enrolment, either as curative or palliative. The most common site for radiation was the supraclavicular region (n = 6).

The mean (± standard deviation [SD]) size of the primary tumor in patients was 31.59 (±21.12) mm. Seven patients presented with a second tumor, with a mean (±SD) size of 20.00 (±10.03) mm. Only one patient presented with a third tumor (size: 8 mm) S1 Table.

Approximately half (51.9%, n/N = 136/262) of early-stage patients were assessed by the study investigator to have an intermediate risk of recurrence (tumor size > 1 cm., node negative) within the next three years. Moreover, 42.4% (n/N = 111/262) of the early-stage patients were judged to be at high risk of recurrence (node positive ≥ 1 node) in the same timeframe.

### Chemotherapeutic treatment and factors governing choice of regimen

Chemotherapy was prescribed in 281 patients (250 [95.4%] early-stage and 31 [100.0%] mTNBC), most commonly in combination with either supportive care (39.1%; n/N = 110/281) or radiotherapy and supportive care (21.7%; n/N = 61/281) or radiotherapy (8.5%; n/N = 24/281). A total of 13.9% (n/N = 39/281) patients received chemotherapy alone.

In early-stage patients, chemotherapy was prescribed as either neoadjuvant (n = 17, 6.8%) or adjuvant therapy (n = 233, 93.2%) Table 2. The most favored adjuvant combination in early-stage TNBC patients was doxorubicin/epirubicin with cyclophosphamide (AC/EC: 23.6%, n/N = 59/250), followed by fluorouracil, doxorubicin/epirubicin, and cyclophosphamide [FAC/FEC/CAF/CEF; 22.4% (n = 56)]. The most favored adjuvant sequential chemotherapy was AC/EC prior to taxanes [AC/EC–≥ paclitaxel (12.4%, n = 31) and AC/EC–> docetaxel (8.4%, n = 21)]. Twelve (4.6%) of the early-stage patients did not receive any chemotherapy, because of non-specified reasons (n = 6), patient’s decision to not receive it (n = 5), and having completed chemotherapy prior to study inclusion (n = 1).

**Table 2:** Chemotherapy in early-stage* TNBC patients.

	Stage I N = 43	Stage II N = 148	Stage III N = 59	Early-stage N = 250
**Neoadjuvant**				
*Combination*				
AC/EC	0 (0.0)	3 (2.0)	6 (10.2)	9 (3.6)
FAC/FEC/CAF/CEF	0 (0.0)	1 (0.7)	3 (5.1)	4 (1.6)
*Sequential*				
AC/EC→Paclitaxel	0 (0.0)	3 (2.0)	0 (0.0)	3 (1.2)
FAC/FEC/CAF/CEF→Docetaxel	0 (0.0)	0 (0.0)	1 (1.7)	1 (0.4)
**Adjuvant**				
*Combination*				
AC/EC	16 (37.2)	35 (23.7)	8 (13.6)	59 (23.6)
FAC/FEC/CAF/CEF	11 (25.6)	39 (26.4)	6 (10.2)	56 (22.4)
Docetaxel+Cyclophosphamide (TC)	7 (16.3)	20 (13.5)	2 (3.4)	29 (11.6)
CMF	6 (14.0)	4 (2.7)	2 (3.4)	12 (4.8)
Docetaxel+AC/EC (TAC/TEC)	0 (0.00)	2 (1.4)	3 (5.1)	5 (2.0)
Gemcitabine+Carboplatin	1 (2.33)	1 (0.7)	0 (0.00)	2 (0.8)
*Sequential*				
AC/EC→Paclitaxel	1 (2.3)	16 (10.8)	14 (23.7)	31 (12.4)
AC/EC→Docetaxel	0 (0.0)	11 (7.4)	10 (17.0)	21 (8.4)
FAC/FEC/CAF/CEF→Paclitaxel	0 (0.0)	1 (0.7)	0 (0.0)	1 (0.4)
FAC/FEC/CAF/CEF→Docetaxel	1 (2.3)	8 (5.4)	3 (5.1)	12 (4.8)
AC→Docetaxel→Paclitaxel	0 (0.0)	2 (1.4)	0 (0.0)	2 (0.8)
AC→Paclitaxel→Capecitabine	0 (0.0)	0 (0.0)	1 (1.7)	1 (0.4)
AC→Paclitaxel→Eribulin	0 (0.0)	1 (0.7)	0 (0.0)	1 (0.4)
*Single agent*				
Capecitabine	0 (0.0)	1 (0.7)	0 (0.0)	1 (0.4)

TNBC–triple-negative breast cancer; A–doxorubicin; C–cyclophosphamide; E–epirubicin; F–fluorouracil; M–methotrexate. Values are presented as n (%) unless specified otherwise

All patients with mTNBC received chemotherapy, either as a combination, or as a single agent, or in a sequential manner Table 3. The most commonly prescribed chemotherapeutic regimen in patients with metastatic TNBC was FAC/FEC/CAF/CEF (32.3%, n/N = 10/31), followed by AC/EC (22.6%, n = 7), and capecitabine (16.1%, n = 5).

**Table 3:** Chemotherapy in metastatic TNBC patients.

	N = 31
*Combination*	
AC/EC	7 (22.6)
FAC/FEC/CAF/CEF	10 (32.3)
Paclitaxel+Cyclophosphamide	1 (3.2)
Paclitaxel+Bevacizumab	1 (3.2)
*Sequential*	
AC→Paclitaxel	1 (3.2)
*Single agent*	
Docetaxel	3 (9.7)
Paclitaxel	3 (9.7)
Capecitabine	5 (16.1)

TNBC–triple-negative breast cancer; A–doxorubicin; C–cyclophosphamide; E–epirubicin; F–fluorouracil. Values are presented as n (%) unless specified otherwise

The choice of chemotherapeutic regimen was categorized according to tumor factors, subject factors, and center factors. The top-ranking factors for each category in early-stage patients were performance status (46.9%, n/N = 123/262) S1 Fig, consensus guidelines in Thailand (44.3%, n/N = 116/262) S3 Fig, and nodal status (32.1%, n/N = 84/262) S2 Fig. Likewise, in case of patients with mTNBC, these were performance status (54.8%, n/N = 17/31), consensus guidelines in Thailand (45.2%, n/N = 14/31) and organ involvement (32.3%, n/N = 10/31), S1–S3 Figs.

### Disease-free Survival (in early-stage patients)

**Fig 1:**
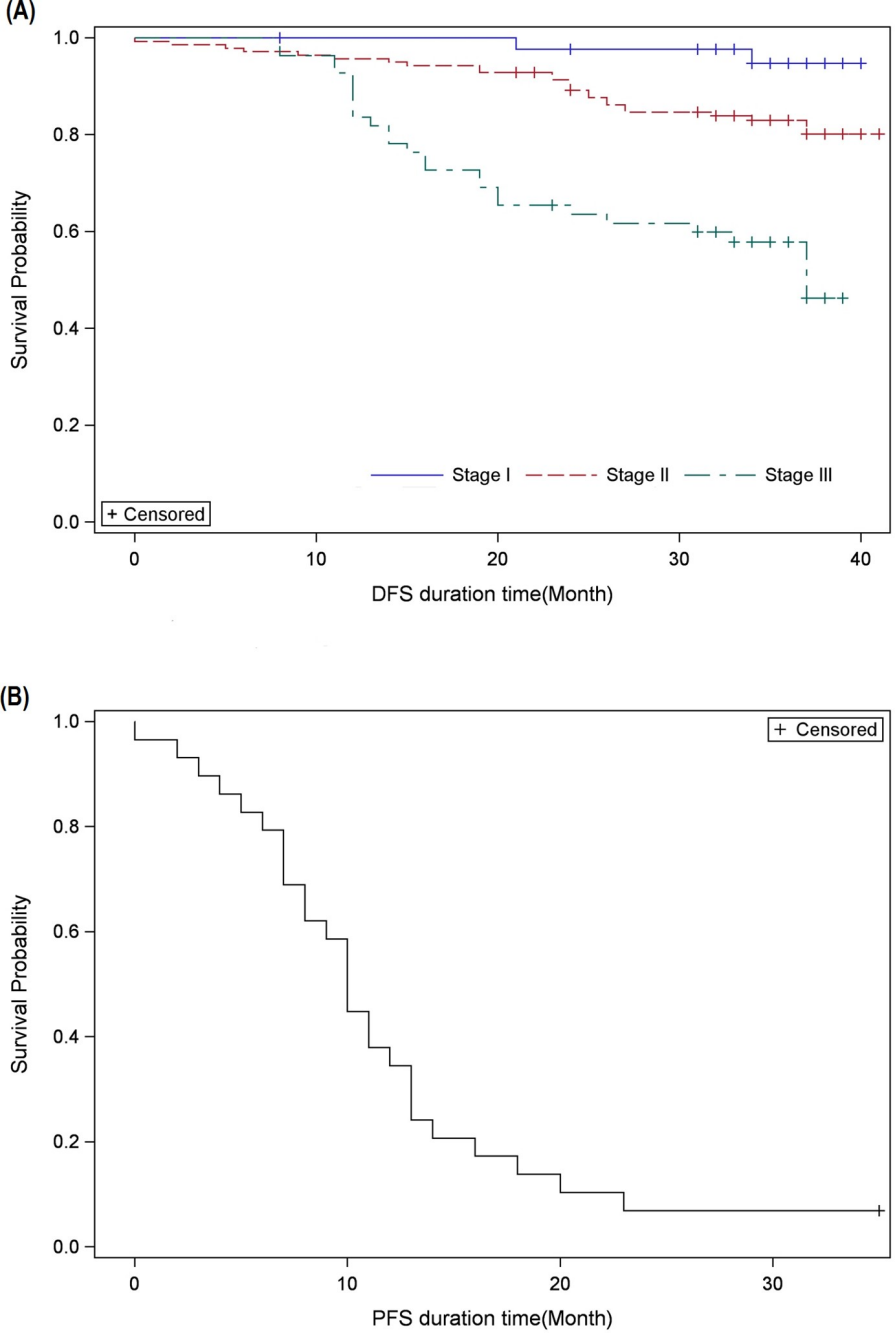
Kaplan-Meier survival analysis for. (A) Disease-free survival (DFS) in early-stage patients and, (B) Progression-free survival (PFS) in patients with metastasis.

At 3-years, 94% of stage I and 80% of stage II patients survived the study without disease, hence, the median DFS could not be computed for these two groups of patients. While for Stage III patients, the median DFS was found to be 37.0 months (Fig 1A).

No statistically significant difference in DFS in early-stage patients was observed when analyzed by factoring in taxane versus non-taxane chemotherapy (p = 0.2931). This pattern was also repeated for sub-group analysis based on tumor staging.

Similarly, no statistically significant difference in DFS in early-stage patients was apparent when analyzed for anthracycline versus non-anthracycline–based chemotherapy (p = 0.9162). Furthermore, this lack of difference in DFS was repeated for Stage I and II patients when probed for anthracycline-based chemotherapy. However, in Stage III patients, median DFS was found to be longer in patients receiving anthracycline-based chemotherapy (DFS 37.0 months vs. 13.5 months in patients who received non-anthracycline-based treatment; p = 0.0181, S4 Fig.

### Progression-free Survival *(*in patients with mTNBC*)*

The median PFS in patients with mTNBC was found to be 10.0 months (IQR: 7.0–13.0 months), Fig 1B.

The PFS was found to be longer in patients who received non-taxane-based chemotherapy in comparison to those who received a taxane-based chemotherapy (12.5 months vs. 8.0 months; p = 0.0025),S5 Fig. No statistically significant difference in PFS was observed in between patients who received anthracycline-based chemotherapy in comparison to those who were administered a non-anthracycline-based chemotherapy.

### Overall survival

**Fig 2:**
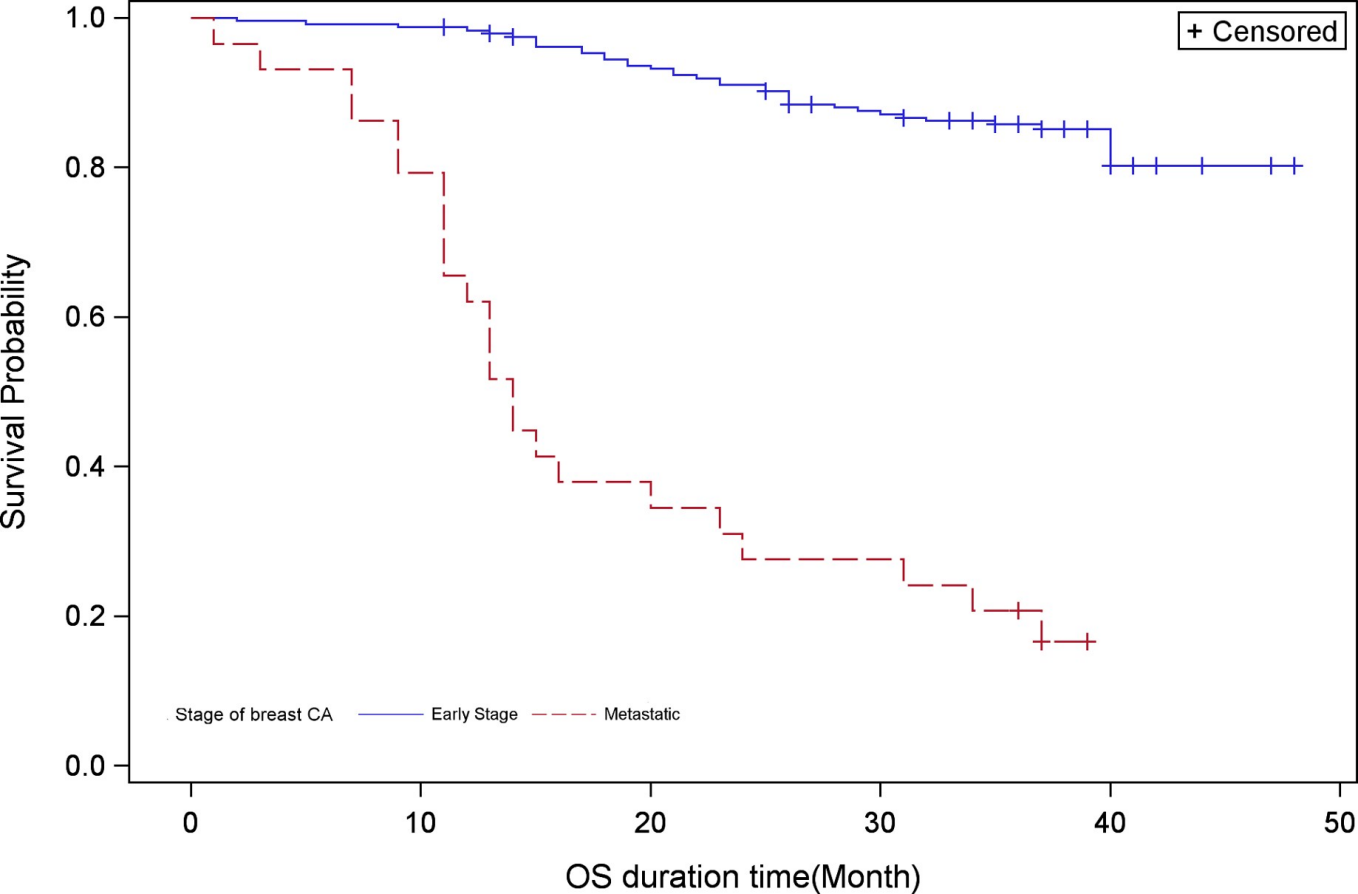
Kaplan-Meier survival analysis for Overall Survival in early-stage and metastatic patients.

The 3-year survival rate in early-stage patients was calculated to be 85.0%. In contrast, this survival rate in patients with mTNBC was 21.0% (Table 4 and Fig 2). Median OS had not been attained in early-stage patients and was calculated as 14.0 months (IQR: 11.0–23.0 months) in mTNBC.

**Table 4:** Overall survival in patients.

	Early-stage	Metastatic	Overall
Overall			
Number of patients	237	29	266
Number of deaths	36	24	60
Follow-up time (in months)			
Median (IQR)	37.0(35.0–39.0)	14.0 (11.0–31.0)	37.0(32.0–38.0)
Min–Max	2.0–48.0	1.0–39.0	1.0–48.0
Median (IQR) survival time (in months)	NA*	14.0 (11.0–23.0)	NA*
1-year OS probability	0.99(0.96–1.00)	0.66(0.45–0.80)	0.95(0.92–0.97)
2-year OS probability	0.91(0.87–0.94)	0.31(0.16–0.48)	0.84(0.80–0.88)
3-year OS probability	0.85(0.80–0.89)	0.21(0.08–0.37)	0.78(0.73–0.83)

IQR–interquartile range; OS–Overall Survival; NA–not available

*Final survival probability of early-stage patients was >50.0% and hence median survival times could not be calculated

15 cases had lost to follow-up/subject withdraw consent and 12 cases did not receive chemotherapy

Among early-stage patients, Stage I and II patients had not reached a median OS; though at the end of the 3-year study period, the overall survival was 100.0% and 90.0%, respectively S6 Fig. Furthermore, subgroup analysis in these patients according to taxane- or anthracycline-based chemotherapy reiterated the general result, i.e., median OS could not be ascertained at study conclusion. In contrast to these patients, median OS in Stage III patients was calculated as 40.0 months S6 Fig. There was a trend of higher median OS in Stage III patients who received taxane-based chemotherapy than non-taxane-based regimen S7 Fig. Also, Stage III patients who received an anthracycline-based regimen were found to have longer OS in comparison to those who received a non-anthracycline-based regimen (40.0 months vs. 16.0 months, p = 0.0027), S8 Fig.

In patients with mTNBC, the median OS was shorter in taxane-based regimen than those who received non-taxane-based regimen (11.00 months vs. 15.50 months; p = 0.3042), although the difference is not statistically significant. In contrast, the median OS in patients with mTNBC who received anthracycline-based regimen was statistically non-significantly longer than that of a non-anthracycline-based regimen (15.00 months vs. 11.50 months; p = 0.7403).

### Effects of taxane add-on to anthracycline-based adjuvant chemotherapy

A total of 14 Stage III patients received anthracycline-based adjuvant chemotherapy and of these, three patients were lost to follow-up or withdrew consent. A total of 30 patients received a taxane add-on sequential or combination to anthracycline-based adjuvant chemotherapy.

**Fig 3:**
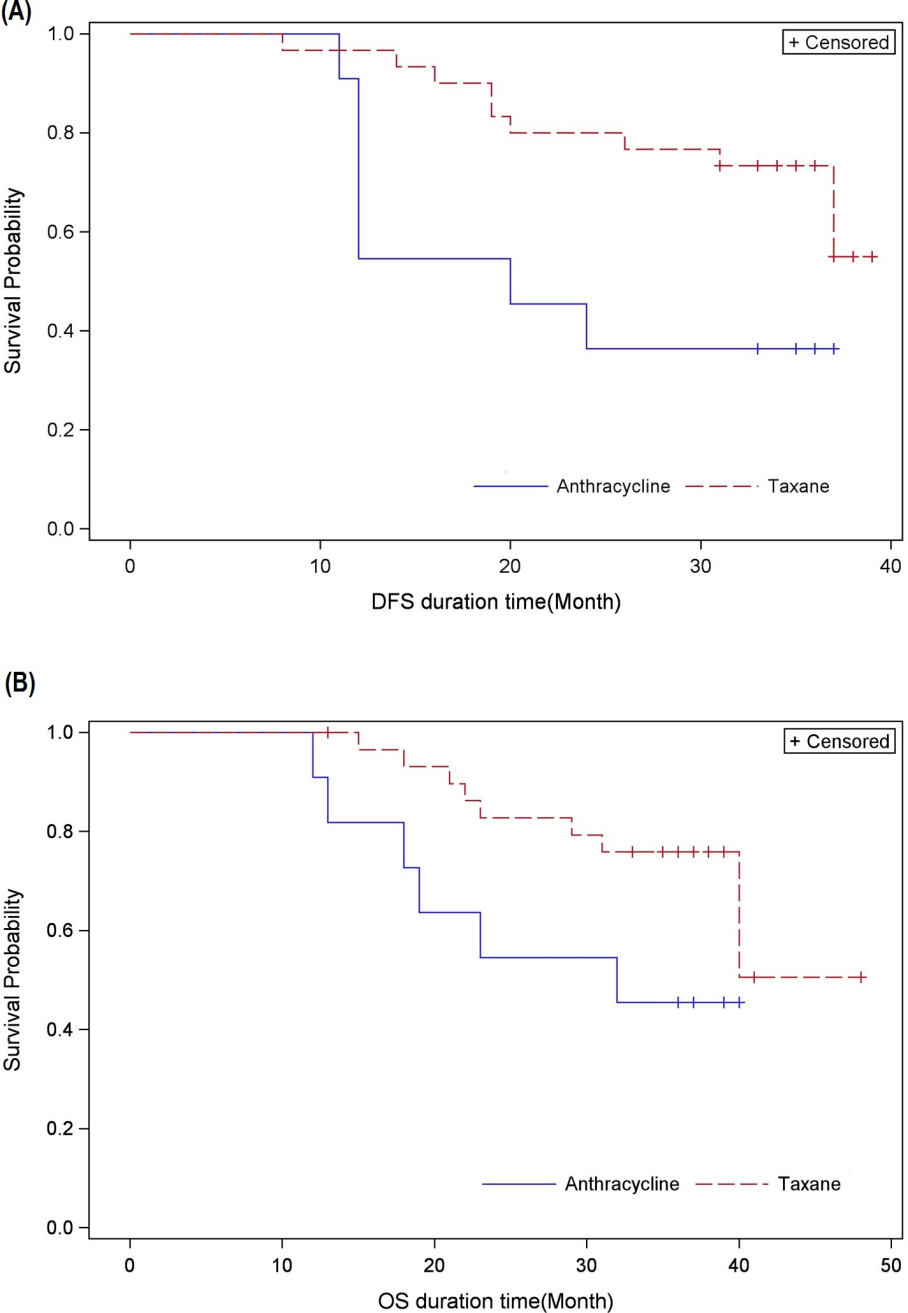
Kaplan-Meier survival analysis for. (A) Disease-free survival (DFS) and, (B) Overall survival (OS) in Stage III patients receiving taxanes either sequentially or in combination with anthracycline-based regimen.

Median DFS and OS in Stage III patients who received only anthracycline-based chemotherapy was calculated as 20.0 months and 32.0 months, respectively (Fig 3). On the other hand, in Stage III patients who had received taxane add-on, median DFS and OS could not be reached since approximately 70% of the patients were alive at the end of study period.

## Discussion

To the best of our knowledge, the reported study is the first of its kind in Thailand to specifically characterize clinical pattern and real-life practices in management of early-stage and metastatic TNBC and assess 3-year survival. Of the 293 patients enrolled in our study, the majority (89.4%) presented early-stage TNBC, mostly Stage II disease. In patients presenting mTNBC, the most common site of metastasis was the lung. Slightly more than half of the early-stage patients were judged to be at a risk for recurrence in three years. Over 95.0% of the patients received adjuvant chemotherapy and anthracycline-based regimens were the adjuvant chemotherapy of choice. During the course of study, survival rate declined with disease progression and was approximately 21.0% in patients with mTNBC in comparison to 100.0% in Stage I patients. Anthracycline-based and non-taxane-based chemotherapeutic regimens were found to have a positive effect on DFS in early-stage TNBC and PFS in mTNBC, respectively.

The lack of receptors that are responsive to hormonal therapy or trastuzumab therapy is a major drawback for the treatment of TNBC [2]. Therefore, currently available options range from surgery in locoregional disease and palliative chemotherapy for progressive TNBC [17]. In our patients, prior surgery was widely reported as first line of treatment; in 91.6% of early-stage patients and 71.0% of patients with mTNBC. However, we could not discern at what stage the choice of surgical intervention in patients mTNBC was made i.e. if surgery was conducted prior to metastasis. Furthermore, total unilateral mastectomy, often without reconstruction, was reported as the commonly used surgical technique.

The current options for adjuvant therapy for early-stage TNBC are anthracycline- and/or taxane-based regimens, usually in combination with cyclophosphamide. Another classical combination that is reportedly active against TNBC is cyclophosphamide + methotrexate + fluorouracil (CMF) [18]. Neoadjuvant anthracycline-based chemotherapy against TNBC is shown to result in pathological complete response and improvement in survival [19]. However, the superiority of anthracycline-based chemotherapy over CMF in an adjuvant scenario is somewhat contentious [20] since conflicting results have been published [21, 22]. In our study, prescription of neoadjuvant chemotherapy was negligible, and the majority of our patients received adjuvant chemotherapy. The most commonly prescribed chemotherapeutic regimens, in both early-stage patients and in patients with mTNBC, were anthracycline-based, either as a combination with cyclophosphamide and/or fluorouracil or as a sequential regimen with taxanes.

Survival rates in patients with TNBC are generally poorer in comparison to those with other breast cancers. Moreover, as in other cancers, a host of independent factors such as tumor stage, molecular composition of the tumor, locoregional advancement, metastatic progression, and choice of treatment govern survival outcomes in TNBC. Hence, it becomes tenuous to compare data gathered in different observational registries. However, any such academic endeavor does provide an overview on real-life outcomes of clinical practices elsewhere. In our study, the 3-year survival rate in early-stage TNBC patients was found to be 85.0%. In contrast, data from the substantially larger California Cancer Registry revealed a 5-year survival rate of 77.0% in patients with TNBC [23]. However, these findings need to be put in perspective, largely because of the shorter time-span of our study and our inability to confirm whether the data from the California Cancer Registry were exclusively for early-stage TNBC patients or if they included mTNBC patients. Of particular interest in our study is the observation on DFS in early-stage TNBC patients: the Stage I and II patients had not achieved median DFS (>50.0% surviving without the disease) wherein median DFS of in Stage III patients was calculated to be 37.0 months. Since the highest probability of disease relapse in TNBC patients is within the first three years after primary treatment [24] and despite a substantial proportion of our early-stage patients were judged to be at high risk of recurrence at enrolment, the observed DFS values are encouraging. In case of patients with mTNBC, we reported an overall PFS of 10.0 months and this was slightly longer in patients who had received non-taxane-based regimens (PFS: 12.5 months). However, when we take into consideration the notable disparity in the numbers of patients who received taxanes or not, it is difficult to draw a conclusion regarding the effectiveness of one regimen over the other.

In our study, median OS in patients with mTNBC was calculated to be 14.0 months and this is comparable to the 9.0–13.3 months OS that has been previously reported [25, 26]. Though not statistically significant, we observed that median OS was longer in those patients with mTNBC who had received a non-taxane-based regimen in comparison to those who had received a taxane-based regimen; and in patients who had received an anthracycline-based regimen in comparison to those who had received a non-anthracycline-based regimen. The PFS was also found to be longer in patients with mTNBC who received non-taxane-based chemotherapy in comparison to those who received a taxane-based chemotherapy. However, our study was not powered enough, i.e. lacked enough patients in each subgroup to establish the validity of this observation. Moreover, patients with mTNBC tended to have received various chemotherapeutic regimens when the preceding one failed to elicit a satisfactory response and hence it is not possible to attribute the net effects to a single regimen.

The use of anthracycline-based chemotherapy with add-on taxanes in neoadjuvant settings has been shown to result in increased pathological complete response in patients with early-stage TNBC [19]. However, the utility of this regimen in adjuvant settings against TNBC has not been convincingly proven. Certain reports have concluded that adjuvant docetaxel or paclitaxel improves DFS and OS [27, 28]. On the other hand, certain studies have failed to recapitulate any advantage to add-on taxanes [29, 30]. We conducted *post hoc* analyses on our study data to probe for any association between survival in Stage III TNBC patients and addition of taxanes to anthracycline-based adjuvant chemotherapy. Our results show a notable improvement in DFS and OS in Stage III patients who had received a taxane sequential to anthracycline-based chemotherapy; median DFS and OS in patients who had received only anthracycline-based chemotherapy were 20.0 and 32.0 months, respectively, while in patients with taxane add-on median DFS and OS had not been reached at the time of study conclusion. These findings are interesting but must be interpreted with caution since the group size was small, therefore, the robustness of the analyses may be limited. Large-scale studies will be required to ascertain whether add-on taxanes to adjuvant chemotherapy have a decided beneficial effect on the treatment of TNBC in Thai settings.

Being an observational study, our investigation was limited in its scope. Survival outcomes in TNBC depend intrinsically on a number of factors including treatment strategy employed. In our study, we could analyze survival data only according to the class of chemotherapeutic agent i.e. taxane versus non-taxane and anthracycline versus non-anthracycline. Hence, we could not identify any particular regimen as more effective than others. Moreover, despite our observed improvements in survival outcomes in early-stage patients who had received anthracycline-based regimen, we advise caution in interpreting these results because of the large proportion of patients who received anthracycline-based treatments. Certain endpoints such as median DFS and OS were not attained for early-stage patients and hence further follow-up was required to identify the impact of current therapeutic practices on survival in Thai patients. However, despite these shortcomings, our study does provide a snapshot of the real-life management of TNBC in Thailand. Our findings highlight the need for further studies to evaluate the effectiveness of contemporary treatment practices in Thailand and could be useful in developing newer and more efficient strategies for treating Thai patients with TNBC.

## Supporting information

S1 Fig Subject factors influencing treatment selection. (TIF)

S2 Fig Tumor factors influencing treatment selection. (TIF)

S3 Fig Center factors affecting treatment selection. (TIF)

S4 Fig Disease-Free Survival of Stage III-subgroup by anthracycline based chemotherapy. (TIF)

S5 Fig Progression Free Survival-subgroup by taxane based chemotherapy. (TIF)

S6 Fig Overall Survival at 3 years-subgroup by early stage patients. (TIF)

S7 Fig Overall Survival of Stage III patients at 3 years-subgroup by Taxane-based chemotherapy. (TIF)

S8 Fig Overall Survival of stage III patients at 3 years-subgroup by Anthracycline based chemotherapy. (TIF)

S1 File (XLSX)

S2 File (XLSX)

S3 File (XLSX)

S1 Table Tumor-specific characteristics. (PDF)
