# Supplementary figures and images for: Real-life clinical pattern, management, and survival in Thai patients with early-stage or metastatic triple-negative breast cancer
Source: PLoS One. 2018 Dec 19;13(12):e0209040. doi: 10.1371/journal.pone.0209040 (PMC6300266; doi:10.1371/journal.pone.0209040)

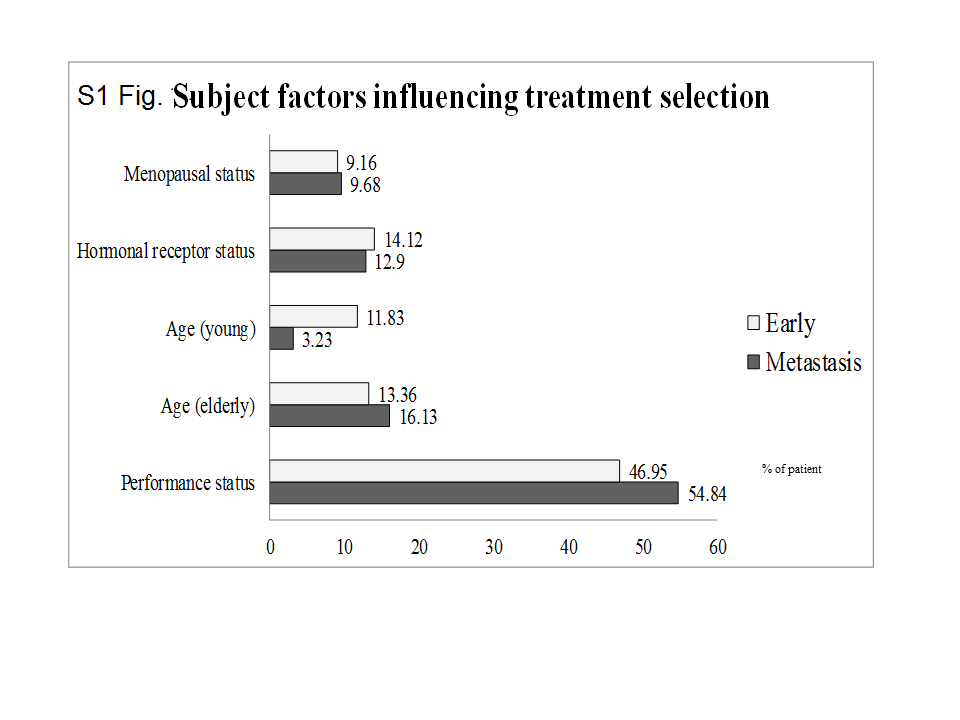

Supplement: S1 Fig — (TIF) [file pone.0209040.s001.tif]

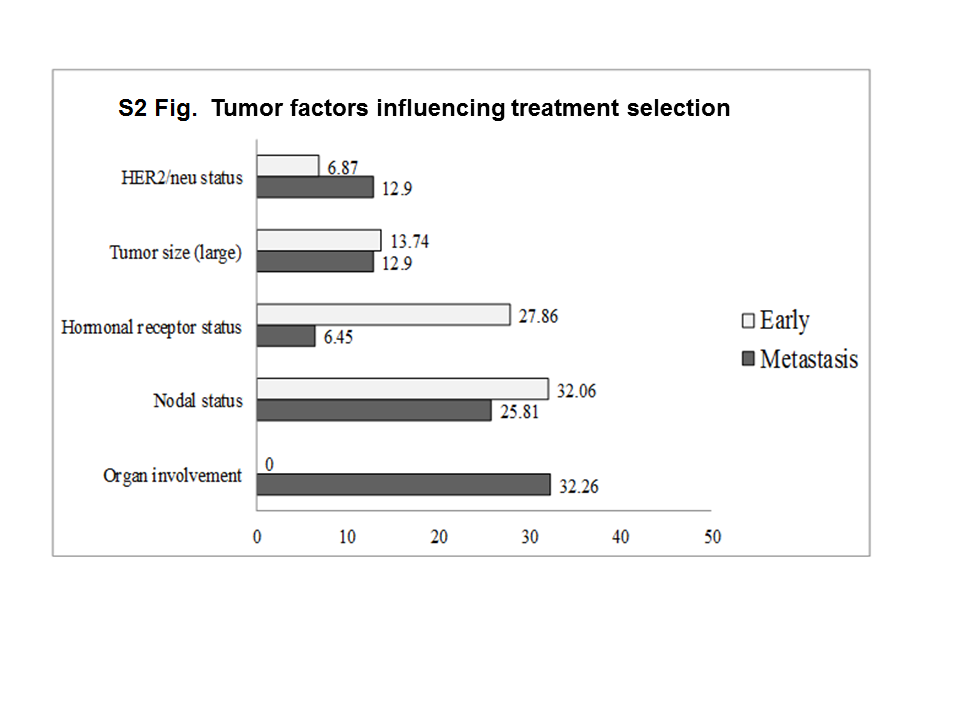

Supplement: S2 Fig — (TIF) [file pone.0209040.s002.tif]

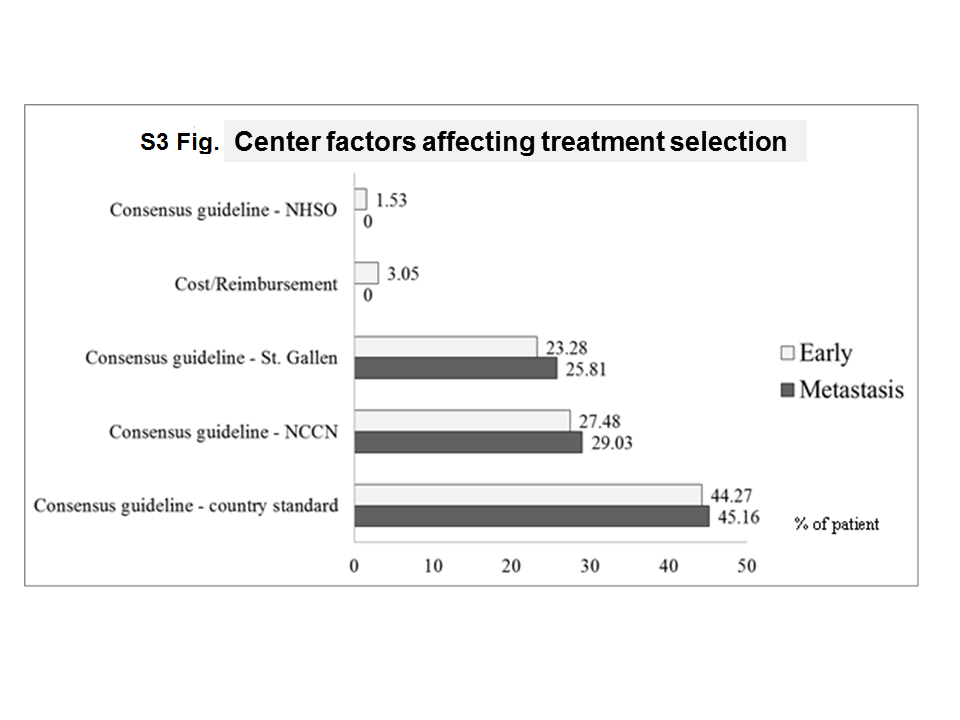

Supplement: S3 Fig — (TIF) [file pone.0209040.s003.tif]

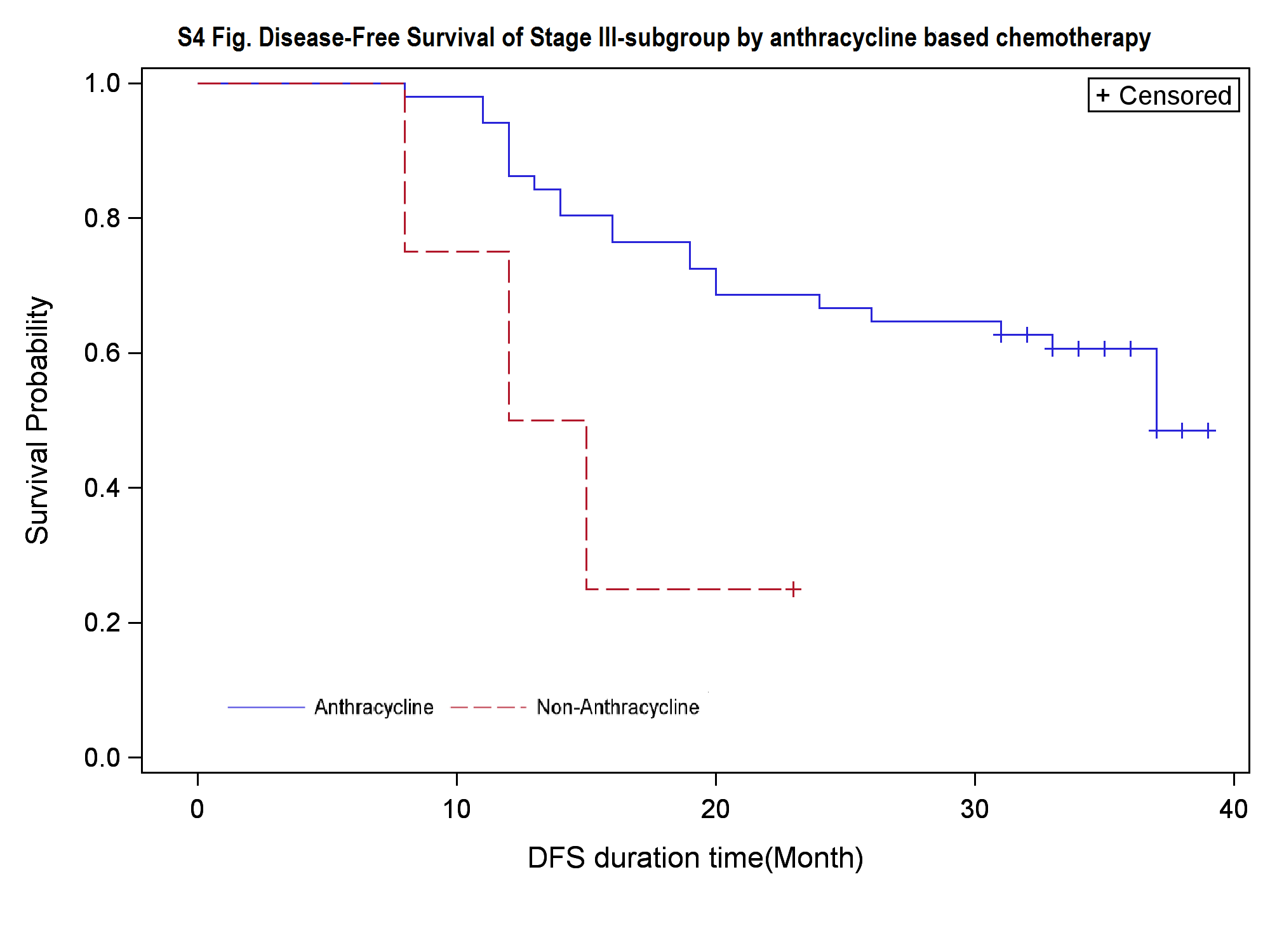

Supplement: S4 Fig — (TIF) [file pone.0209040.s004.tif]

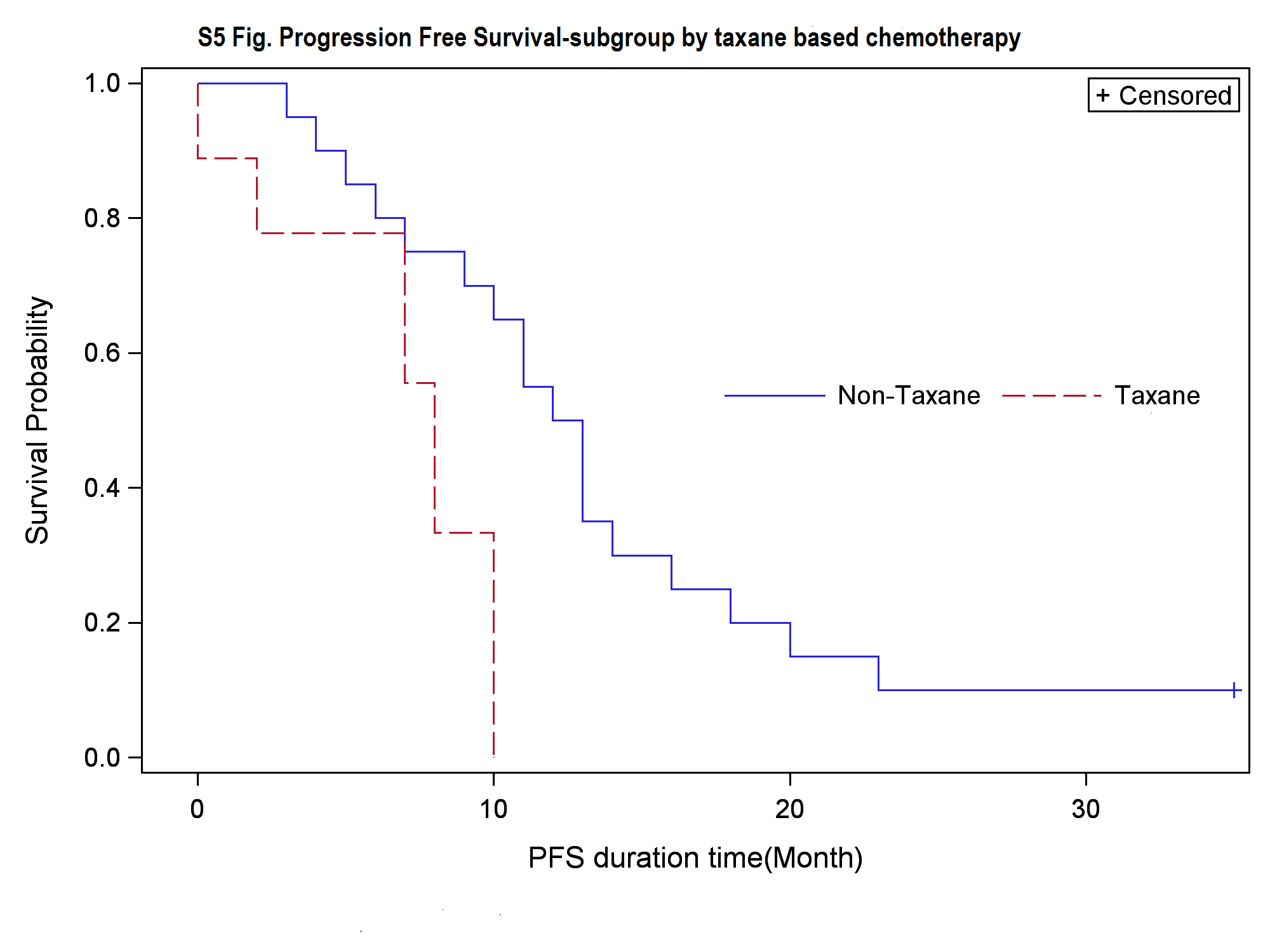

Supplement: S5 Fig — (TIF) [file pone.0209040.s005.tif]

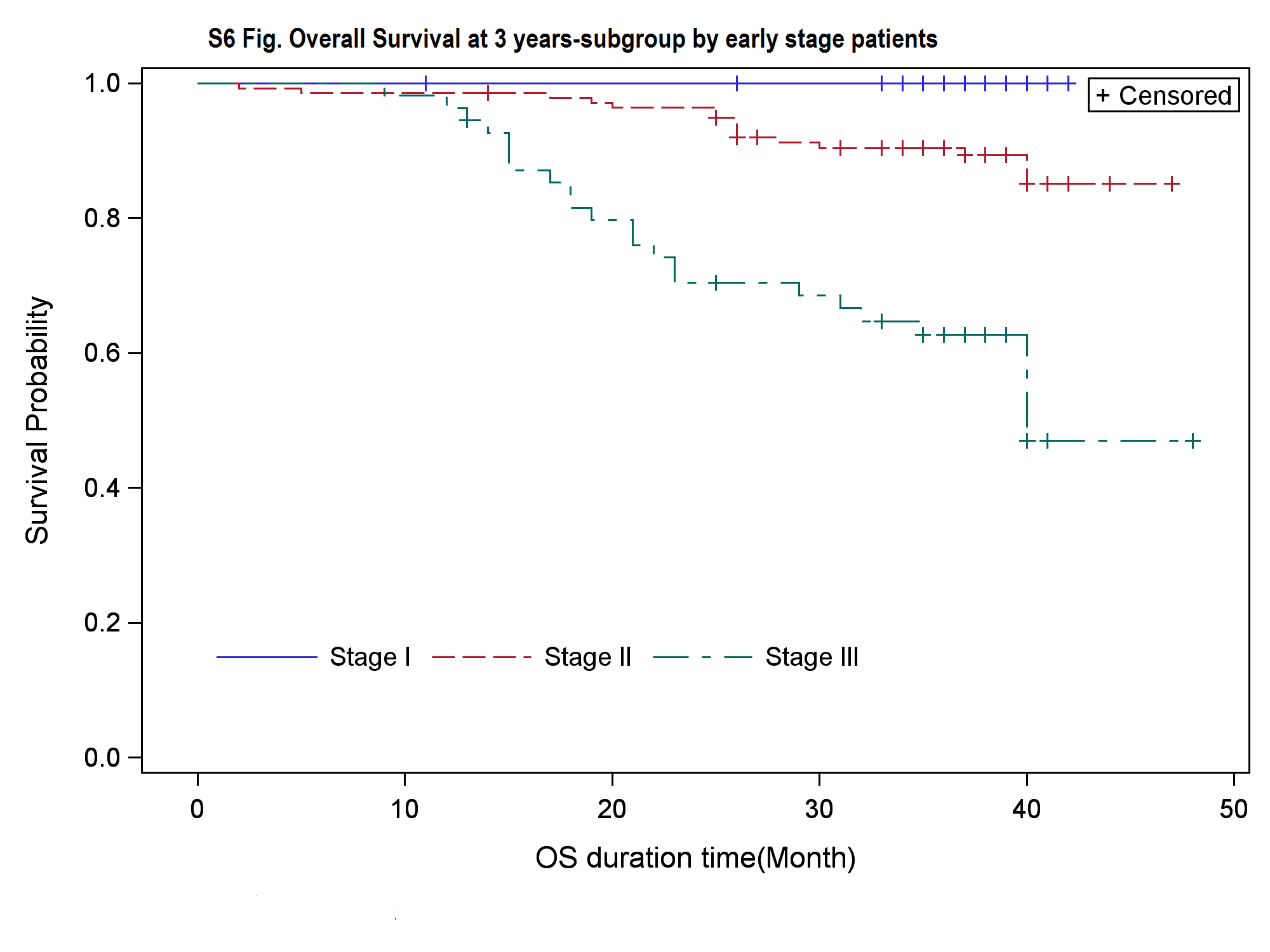

Supplement: S6 Fig — (TIF) [file pone.0209040.s006.tif]

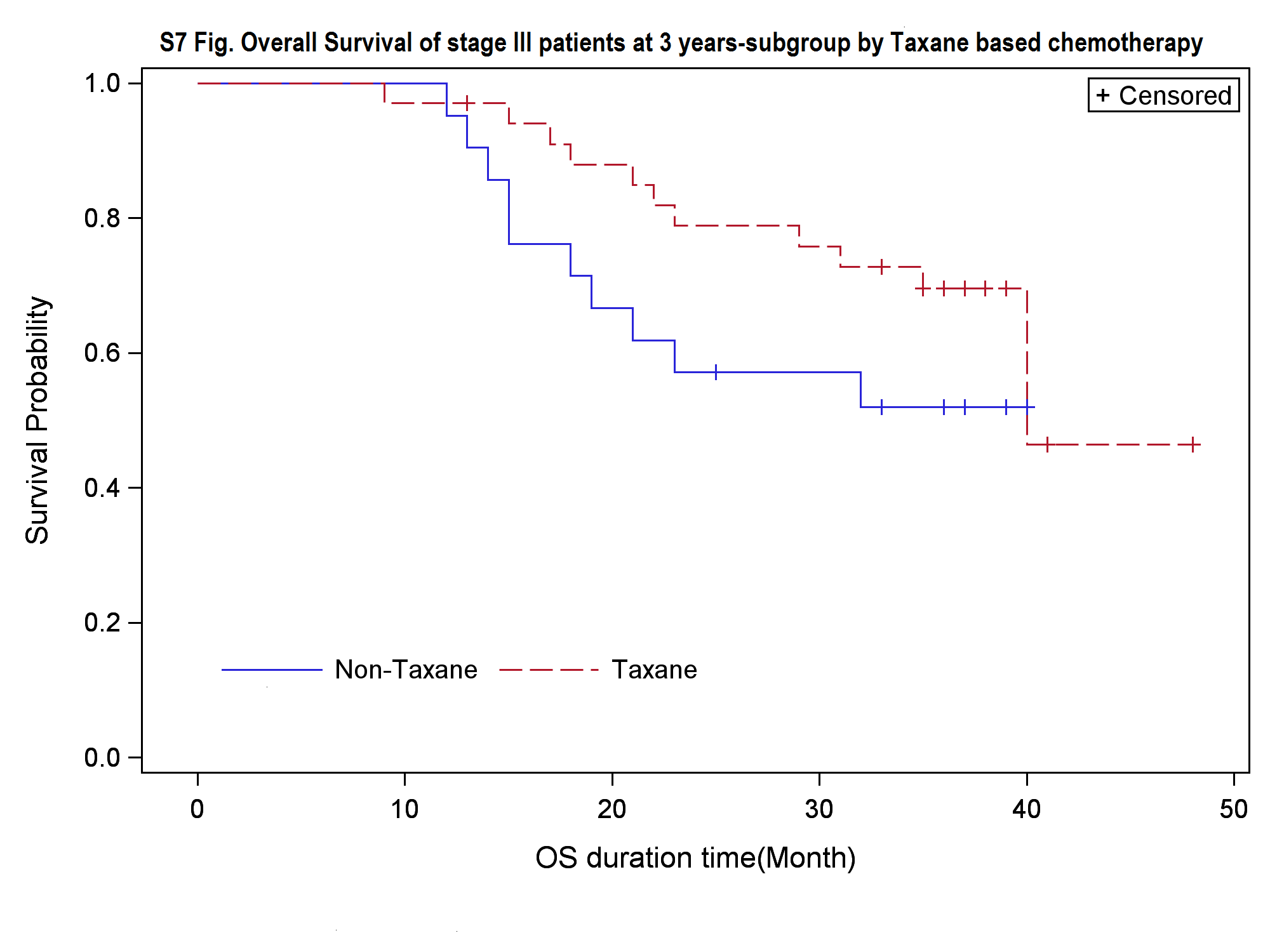

Supplement: S7 Fig — (TIF) [file pone.0209040.s007.tif]

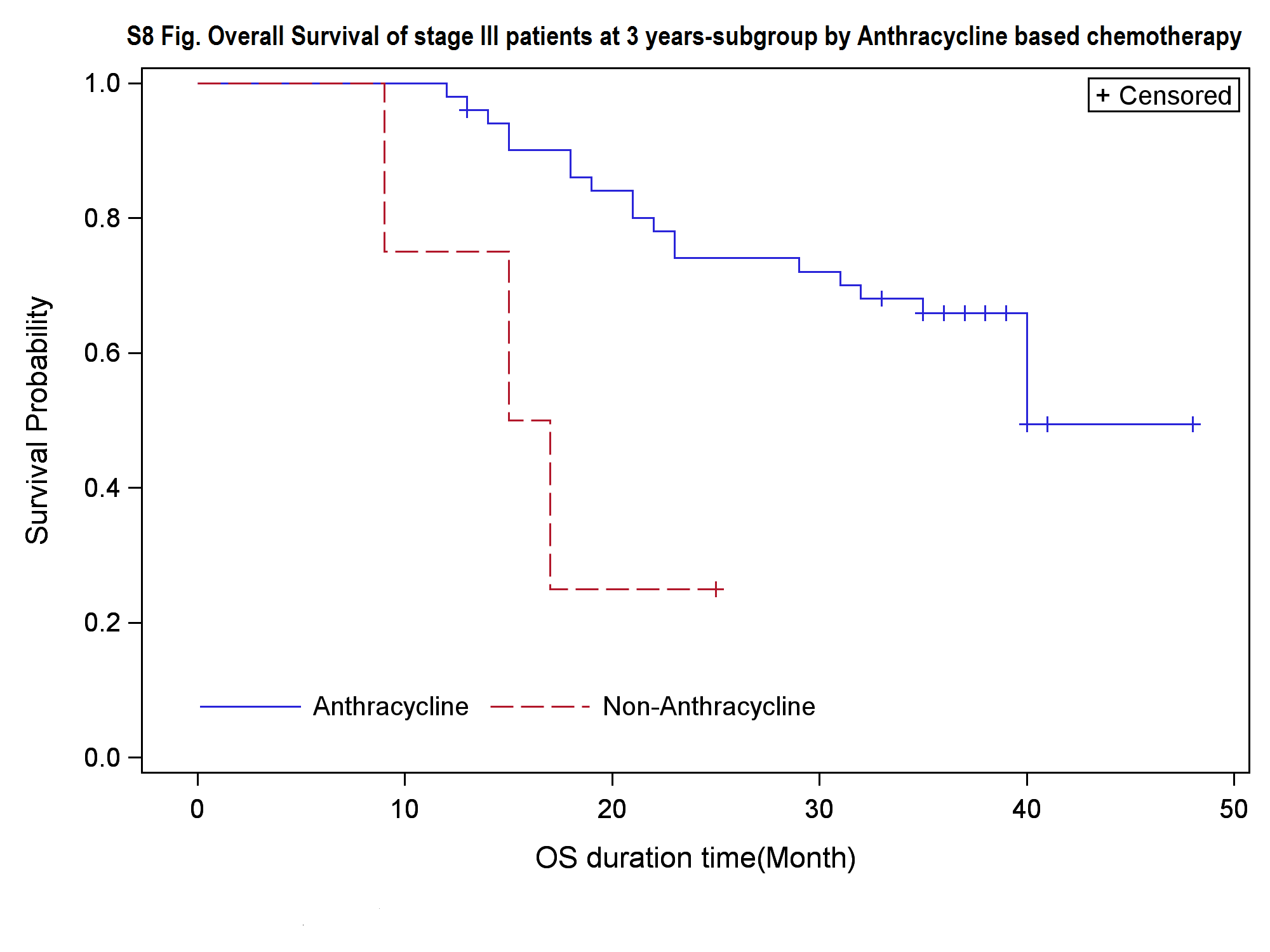

Supplement: S8 Fig — (TIF) [file pone.0209040.s008.tif]
