# Supplementary material for: Real-life clinical pattern, management, and survival in Thai patients with early-stage or metastatic triple-negative breast cancer
Source: PLoS One. 2018 Dec 19;13(12):e0209040. doi: 10.1371/journal.pone.0209040 (PMC6300266; doi:10.1371/journal.pone.0209040)
Supplement: S1 Table — (PDF) [file pone.0209040.s012.pdf]

S1 Table. Tumor-specific characteristics.

Histopathological grade at initial diagnosis	Stage I (N=46)	Stage II (N=151)	Stage III (N=65)	Early (N=262)	Metastasis (N=31)	Overall (N=293)
Grade cannot be assessed	1 (2.17)	8 (5.30)	0 (0.00)	9 (3.44)	14 (45.16)	23 (7.85)
Well differentiated	2 (4.35)	8 (5.30)	3 (4.62)	13 (4.96)	0 (0.00)	13 (4.44)
Moderate differentiated	19 (41.30)	59 (39.07)	30 (46.15)	108 (41.22)	6 (19.35)	114 (38.91)
Poorly differentiated	24 (52.17)	75 (49.67)	31 (47.69)	130 (49.62)	11 (35.48)	141 (48.12)
**1st tumor size (mm)**						
No. of patients	46	141	53	240	20	260
Mean(SD)	16.50 (7.79)	32.10 (15.87)	43.58 (30.13)	31.65 (20.79)	30.90 (25.29)	31.59 (21.12)
Median(IQR)	17 (12.00–20.00)	30 (24.00–40.00)	35 (25.00– 55.00)	26.50 (20.00–40.00)	25 (16.00– 36.50)	25.50 (20.00–40.00)
Min/Max	3.00–60.00	5.00–150.00	5.00–170.00	3.00–170.00	3.00–120.00	3.00–170.00
**2nd tumor size (mm)**						
No. of patients	0	7	0	7	0	7
Mean(SD)	0.00 (0.00)	20 (10.03)	0.00 (0.00)	20 (10.03)	0.00 (0.00)	20 (10.03)
Median(IQR)	0.00 (0.00–0.00)	20(10.00– 25.00)	0.00 (0.00–0.00)	20 (10.00– 25.00)	0.00 (0.00– 0.00)	20 (10.00– 25.00)
Min/Max	0.00–0.00	9.00–38.00	0.00– 0.00	9.00–38.00	0.00–0.00	9.00–38.00
**3rd tumor size (mm)**						
No. of patients	0	1	0	1	0	1
Mean(SD)	0.00 (0.00)	8 (0.00)	0.00 (0.00)	8 (0.00)	0.00 (0.00)	8 (0.00)
Median(IQR)	0.00 (0.00– 0.00)	8 (8.00– 8.00)	0.00 (0.00– 0.00)	8 (8.00– 8.00)	0.00 (0.00– 0.00)	8 (8.00– 8.00)
Min/Max	0.00–0.00	8.00–8.00	0.00–0.00	8.00–8.00	0.00–0.00	8.00–8.00

IQR: Inter-quartile range; SD: standard deviation
